# Leveraging Temporal Trends for Training Contextual Word Embeddings to Address Bias in Biomedical Applications: Development Study

**DOI:** 10.2196/49546

**Published:** 2024-10-02

**Authors:** Shunit Agmon, Uriel Singer, Kira Radinsky

**Affiliations:** 1 Department of Computer Science Technion—Israel Institute of Technology Haifa Israel

**Keywords:** natural language processing, NLP, BERT, word embeddings, statistical models, bias, algorithms, gender

## Abstract

**Background:**

Women have been underrepresented in clinical trials for many years. Machine-learning models trained on clinical trial abstracts may capture and amplify biases in the data. Specifically, word embeddings are models that enable representing words as vectors and are the building block of most natural language processing systems. If word embeddings are trained on clinical trial abstracts, predictive models that use the embeddings will exhibit gender performance gaps.

**Objective:**

We aim to capture temporal trends in clinical trials through temporal distribution matching on contextual word embeddings (specifically, BERT) and explore its effect on the bias manifested in downstream tasks.

**Methods:**

We present TeDi-BERT, a method to harness the temporal trend of increasing women’s inclusion in clinical trials to train contextual word embeddings. We implement temporal distribution matching through an adversarial classifier, trying to distinguish old from new clinical trial abstracts based on their embeddings. The temporal distribution matching acts as a form of domain adaptation from older to more recent clinical trials. We evaluate our model on 2 clinical tasks: prediction of unplanned readmission to the intensive care unit and hospital length of stay prediction. We also conduct an algorithmic analysis of the proposed method.

**Results:**

In readmission prediction, TeDi-BERT achieved area under the receiver operating characteristic curve of 0.64 for female patients versus the baseline of 0.62 (*P*<.001), and 0.66 for male patients versus the baseline of 0.64 (*P*<.001). In the length of stay regression, TeDi-BERT achieved a mean absolute error of 4.56 (95% CI 4.44-4.68) for female patients versus 4.62 (95% CI 4.50-4.74, *P*<.001) and 4.54 (95% CI 4.44-4.65) for male patients versus 4.6 (95% CI 4.50-4.71, *P*<.001).

**Conclusions:**

In both clinical tasks, TeDi-BERT improved performance for female patients, as expected; but it also improved performance for male patients. Our results show that accuracy for one gender does not need to be exchanged for bias reduction, but rather that good science improves clinical results for all. Contextual word embedding models trained to capture temporal trends can help mitigate the effects of bias that changes over time in the training data.

## Introduction

### Background

Word embeddings are machine-learning models that aim to represent words as real numbered vectors. To train the embeddings, a large text corpus is needed. Contextualized word embeddings such as BERT [1], where the representation of a word depends on its surrounding words, have an immense impact on performance in various natural language processing (NLP) tasks. In the clinical domain, embeddings pretrained on clinical texts can be used to perform biomedical NLP tasks [2] or predict clinical outcomes for patients [3]. However, if the training corpus contains biases, they may be perpetuated by the embedding model, and affect the performance on downstream tasks [4-6]. Zhang et al [3] show that word embeddings trained on clinical texts cause performance gaps for different genders and races on clinical tasks.

Clinical trials are the main method to evaluate the efficacy of new treatments on patients, but they may contain biases [7]. For decades, clinical trials excluded women participants [8,9]. The reported reasons for this exclusion include uncertainty about the effects of the menstrual cycle on trial results [10] and tragedies that occurred during trials. For instance, after the thalidomide clinical trial, women of childbearing age were excluded from early-phase clinical trials [8]. Underrepresentation of women leads to a misunderstanding of how women respond to various drugs, which ultimately leads to more adverse drug reactions than in men [11-13]. To mitigate such phenomena, in 1993 the US Food and Drug Administration mandated the inclusion of women in trials [8]. Nevertheless, unequal representation of women persists. Clinical results are not well analyzed nor reported for the influence of gender [9,14].

However, women’s representation in clinical trials significantly improves over time due to constant social and legislative efforts [8]. In a comprehensive study of over 43,000 clinical trial papers from PubMed [9], the representation of women in 11 disease categories was analyzed. They found that the number of women participants from before 1993 until 2018 grew in 6 categories and was unchanged in 3 more. In the remaining 2 categories, the female participant proportion was traditionally higher than the female prevalence—the proportion of female patients out of all patients with the disease. The decrease indicates that the proportion grew closer to the actual female prevalence. They find that in all the categories combined, women’s representation became more accurate. As women’s representation improves, discoveries can be less biased toward women, as reflected in changes in relations between concept embeddings over time (Multimedia Appendix 1).

### Related Work

Existing methods to remove representational gender bias from word embeddings aim to remove sensitive information, for example, gender, from the embeddings using data augmentation [15,16], in-training methods modifying the training objective [17], or posttraining methods such as projections to subspaces [4,18,19]. Recently, adversarial training [3,20,21] was also applied to remove information about protected attributes, for example, gender or race, from the representations. These methods aim for a notion of fairness named demographic parity [22]: an independence between a model’s prediction and the protected attribute. Indeed, a decision model cannot use the protected attribute if it is not recoverable from the embeddings.

However, in the clinical domain, demographic parity should not be applied, since the sensitive attribute (eg, gender) is an important feature in clinical prediction tasks. Therefore, unlike previous works about adversarial debiasing, we do not remove gender information from the embeddings. Instead, we harness the temporal trend of women’s inclusion that exists in the corpus of clinical trials to improve the information captured in the embeddings regarding women.

Another relevant work [23] explored a method where abstracts were weighted by the number of women who participated in the trial to train gender-sensitive Word2vec [24] embeddings. In this work, we aim to explore the benefits of the improvement in female inclusion over time as an alternative method for debiasing. We compare our work to the method in the study by Agmon et al [23] in Multimedia Appendix 2.

The term “temporal distribution matching” was recently used [25] in an entirely different context: time series forecasting, where given a series of samples and their labels over time, a function from samples to labels is learned. Temporal distribution matching in the context of time series forecasting is a method to handle temporal covariate shifts that harm the performance of the learned prediction model. The method is composed of two phases: (1) detecting the different time periods through “temporal distribution characterization” and (2) performing distribution matching on the hidden states of a recurrent neural network model which is the prediction model. To perform the distribution matching, a loss term is added to the model optimization, based on a pairwise distance between the hidden states of the recurrent neural network after consuming each time period of the series. There are 2 main reasons why this method is not applicable to our problem. First, the task is inherently different: we are interested in learning a word representation model, which is an unsupervised task, while the study by Du et al [25] focuses on time series forecasting, which is a supervised task that requires labels. Second, to calculate a loss term such as was introduced in the study by Du et al [25] requires comparing the state of an embedding model after reading all texts from each time period; embedding models usually do not support such a long context in a meaningful way. Instead, our method uses an adversary component to perform the distribution matching while only looking at 1 abstract at a time. Our method can be viewed as an adjustment of temporal distribution matching to the task of word representation learning.

### Goal of This Study

One method to use the improvements in clinical trial practices is to repeat past clinical trials using the new practices. However, it is not a feasible option due to both ethical concerns and the costs of clinical trials. From the machine learning point of view, a naive solution would be to train the embedding model only on the more recent papers; but such a model is trained on far less data. This may yield to suboptimal performance on downstream tasks. We aim to train word embeddings that (1) make use of the entire data set of clinical trial abstracts, (2) harness the positive temporal trends in clinical trials, and (3) achieve high performance on the downstream tasks for the underrepresented group.

Intuitively, we would like to match the distribution of earlier clinical findings to that of more recent findings. We present TeDi-BERT—a temporal distribution matching training method, applied to BERT word embeddings. In this method, in parallel with the original training process of the embeddings, an adversarial temporal classifier tries to distinguish old from new samples based on their embeddings, while the embedding model tries to *decrease* the adversary’s performance. Intuitively, if the temporal classifier’s performance is low, then the embeddings of older clinical trials are similar to those of more recent clinical trials. The competition between the embedding model and the temporal classifier acts as a temporal distribution matching mechanism. We use the adversarial component because adversarial models were successfully applied in domain adaptation [26], which is similar to our setting: the different time periods can be viewed as 2 domains.

While there are methods to tackle model biases directly, in this work we explore the effects of temporal distribution matching on bias. Additionally, the proposed method can capture a wide range of trends, such as the emergence of new diseases and new practices. However, in this work, we focus on evaluating its effects on gender bias. Although the method is generic, gender bias is a real practical problem, where temporal trends have been present for years [9]. Evaluating other aspects of temporal distribution matching is left for future work.

We evaluated the model on several tasks, including clinical tasks, based on the MIMIC-III data set [27], and compared the performance on female and male patients.

We contributed our code and data sets [28] to the community to be leveraged for additional tasks where subpopulations are underrepresented.

## Methods

### Overview

A word embedding is a mapping from words to real numbered vectors, such that the vector captures the meaning of the word. Word embeddings are usually trained on a large corpus of text, using a semantic task. For example, in BERT [1] embeddings, some words in the sentence are masked, and the word vectors of the remaining words are used to predict the masked words. The loss from this prediction task is then used to tune the word vectors: the word representations are modified to better perform the task.

In this work, we describe TeDi-BERT, a temporal distribution matching training method, applied to BERT. We trained the word embeddings on PubMed abstracts of clinical trials between 2010 and 2018. We focused on this time range because there were much fewer clinical trials in ClinicalTrials.gov before 2010, and we used ClinicalTrials.gov to filter the clinical trial abstracts.

One could argue that a better data set to use for training is EHR data, such as the medical notes from MIMIC-III. Numerous factors contributed to our decision not to pursue that course of action. The first is a technical reason: the timestamps available in MIMIC-III were randomly shifted to preserve patient privacy, so visits from different patients are not guaranteed to be in the correct order. Second, the practices and methods in these medical notes represent the conventions used in a single place of medical care, unlike clinical trials which are more diverse, and cover practices and methods from different geographic places. Finally, to validate our choice of training data set, we conducted a qualitative analysis of the trends that exist in clinical trial abstracts and found several examples of real-world trends that were quickly reflected in clinical trial abstract data (Multimedia Appendix 3).

To harness the temporal trends in these clinical trials, we require that the distribution of embeddings of the older abstracts be similar to that of newer abstracts. In addition to training the embedding model on the original semantic task, we simultaneously train it on a temporal classification task.

**Figure 1:**
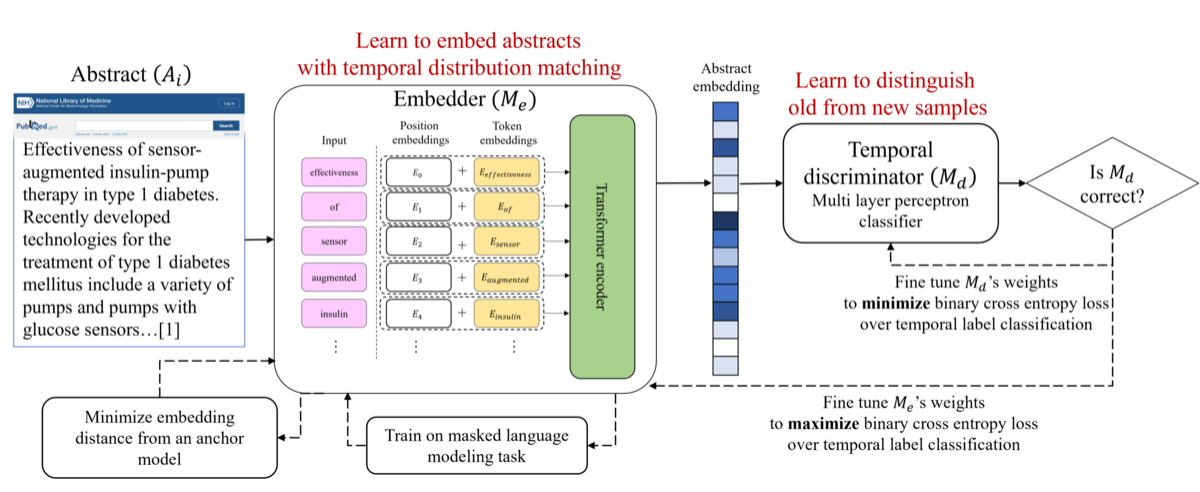
Schematic drawing of the TeDi-BERT model for health care embeddings. Clinical trial abstracts are embedded using a BERT model, and a discriminator aims to distinguish between old and new abstracts. The embedder simultaneously trains on the original embedding task of masked language modeling and regulates the embeddings to resemble an anchor model. TeDi-BERT: temporal distribution matching applied on BERT.

The abstracts were divided into old, 2010-2013, and new, 2016-2018 (see below for details on the choice of time ranges), and assigned a temporal label. A temporal discriminator, namely, a classifier, aims to distinguish old from new abstracts based on their embeddings. The embedding model, however, aims to *reduce* the classifier’s performance by tuning the embeddings. To translate this idea into an architecture (Figure 1), we leveraged the well-received framework of generative adversarial networks (GANs) [29], where 2 components (a generator and a discriminator) compete on a task with opposite goals.

For example, an abstract from 2010 is transformed into a vector representation using the BERT embedder. The embedding vector is fed to the temporal discriminator. Assume that the discriminator correctly predicted that this sample is “old” with probability *p*. The discriminator’s weights are then updated so that *p* is closer to 1, while the embedder’s weights are updated so that *p* is closer to 0.

The embedding model (*M*_e_) is given an abstract, performs the semantic prediction task on the abstract text, and computes the semantic loss (*L_MLM_*). Additionally, the same embedding model acts as the generator in the GAN and emits an embedding for the full abstract.

The abstract embedding is fed to the temporal discriminator (*M_d_*), which is a classifier trying to distinguish whether the embedding belongs to a new or old abstract. A binary cross entropy loss (*L_adv_*) for this task is computed using the discriminator output and the temporal label. The discriminator aims to minimize this loss. However, the generator aims to both maximize the loss and simultaneously minimize *L_MLM_*.

Consider a trivial generator that outputs the same embeddings regardless of the input text. In this case, the discriminator cannot distinguish old from new texts, and *L_adv_* would be minimized. To prevent such cases, we wish the model to preserve the original semantics of the texts. We therefore added another term to the loss function, which was meant to anchor the embedding model, so that it did not drift too far from the original embedding. We embed each sample using a frozen anchor model and compute the loss term (*L_A_*) as the L2 Frobenius norm distance between the frozen embedding and the generator’s embedding. The final objective function is given by:



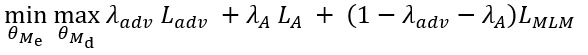



Where *θ_M_* denotes the parameters of a model *M*, and *λ_adv_* and *λ_A_* are hyperparameters used to balance the different components.

### Implementation Details

The corpus of clinical trial abstracts from 2010 to 2018 was divided into old (2010-2013) and new (2016-2018) clinical trials. The guiding principle in choosing these time ranges is to create a gap between the 2 time periods, while maintaining a large enough and balanced number of abstracts in each set. The first time range is 1 year longer since there are less abstracts per year in 2010-2013 (~5000 on average) versus 2016-2018 (~9000 on average). The gap is needed for the discriminator task: it is harder to distinguish between abstracts from consecutive years since the temporal trends are slow. When comparing the 2 time ranges, we observed a statistically significant increase over time in the percentage of women participants in clinical trials (Multimedia Appendix 1). This is consistent with previous findings [9] over slightly different time ranges: the total enrollment bias for women was improved from before 1993 (–0.11) to 2014-2018 (–0.05).

As the embedding model, we chose BERT [1], a transformer-based model for contextualized word embeddings. We used a small version of BERT, named BERT-tiny [30], with 2 transformer layers and a hidden representation size of 128, pretrained on BookCorpus [31] and the English Wikipedia. Smaller models require less computation resources and are therefore more affordable and accessible. Rosin et al [32] have shown that BERT-tiny–based models were comparable to BERT-base in their ability to learn temporal trends. We witnessed a similar phenomenon on the clinical task of length of stay (LOS) prediction (Multimedia Appendix 4).

We initialized the model from a version of BERT which was not trained on any scientific or medical data, so that we could attribute the medical knowledge accumulated in the model only to the clinical trial abstracts in the corpus used in the train set.

As each abstract is long, and BERT has a maximal input length of 512-word pieces, we split it into sentences using the Natural Language Toolkit tokenizer [33]. The generator embeds each sentence. The first *m* sentence embeddings are concatenated and fed to the discriminator, which is a linear classifier. Hence the classifier size is *d* ⋅ m+1. As 96.97% (21123/21784) of abstracts had up to 20 sentences, we set *m* = 20 and padded shorter abstract embeddings with zeros before feeding them to the discriminator. As a frozen anchor model, we used a BERT model of the same architecture as the generator, initialized similarly but trained only with masked language modeling (MLM) on all of the abstracts.

The embedder and discriminator components of TeDi-BERT were trained simultaneously, 1 batch at a time for 20 epochs. Each component was optimized using the Adam optimizer with a learning rate of 2*e*–5. Additional technical details are given in Multimedia Appendix 5.

The TeDi-BERT model used in our experiments was trained with *λ_adv_*=0.3, *λ_A_*=0.3, hence the weight of the *L_MLM_* term was 0.4. We experimented with *λ_adv_,λ_A_*∈{0,0.1,…,0.6} and chose the best combination according to the model’s ability to predict the future semantic relatedness of medical concepts (Section S3 in Multimedia Appendix 6).

### Experimental Evaluation Setup

The corpus used to train the embedding models is composed of PubMed [34] abstracts describing clinical trials on humans. To select only those abstracts out of the 90,000 available in PubMed version of 2020, we match each abstract with an entry from ClinicalTrials.gov [35] according to the NCT identifier inside the abstract text, leaving 21,784 abstracts, 12,452 of them from 2010-2013 and 2016-2018. We randomly split the data into 70.51% (8780 abstracts) train and 29.49% (3672 abstracts) test, and kept this partition fixed throughout our experiments.

For our downstream tasks, we used 2 different clinical prediction tasks, created based on the MIMIC-III data set [27], an anonymized and publicly available data set that contains information about patients at a massive tertiary care hospital. The data set contained 58,976 hospital admissions with 61,532 intensive care unit (ICU) stays over 46,520 distinct patients. After removing patients aged younger than 18 years (as performed in the study by Lin et al [36]), 38,552 patients remained. We randomly divided the patients into train and test sets, so that data from a single patient could not appear in both the train and the test. The train set contained 30,817 patients, out of which 43.97% (n=13,553) were female, and the test set contained 7735 patients, out of which 43.33% (n=3352) were female.

### Downstream Tasks

*LOS prediction*—a regression task predicting a patient’s LOS in the hospital in days. Predicting LOS is a common clinical task, which is important in hospital resource allocation planning. The predictions can also be taken as indications of the severity and need for different levels of care and recovery.

To predict the LOS we used the patient’s diagnoses from their previous admissions, and the primary diagnosis from the current admission, along with demographic features and summary features (number of previous admissions, procedures and diagnoses, and time since the last admission).

*Readmission prediction*—a classification task predicting unplanned ICU readmission of a patient, at the time of their discharge. Such readmissions indicate an unexpected deterioration in the patient’s state. Detecting such cases in advance can improve the quality of care for the patients by allocating special programs and resources that address reasons for readmission. We followed Lin et al [36] for the definition of unplanned readmission: patients that were transferred from the ICU to low-level wards or discharged, but returned to the ICU or died within 30 days. The features used in this prediction task are the patient’s diagnoses from previous admissions, and diagnoses and medications from the current admission (which are known at the time of discharge), along with demographic features.

### Compared Models

We compared the following models in our experiments:

*Nonmedical BERT*—a pretrained BERT on English Wikipedia and BookCorpus, not trained on any clinical data [30].

*Medical BERT 2010-2018*—this baseline represents the natural way to train BERT for clinical uses: training BERT with the MLM task over the clinical texts. The model was initialized with nonmedical BERT and trained for 40 epochs on clinical trial abstracts between 2010 and 2018.

*Null it out [18]*—As an example of a debiasing method aiming to remove gender information from the embeddings, we applied the method presented in the study by Ravfogel et al [18] on medical BERT 2010-2018. This method was found to be best at debiasing BERT embeddings to remove gender stereotypes [37]. The method is based on iterative null space projection of the embeddings so that the sensitive information (gender) cannot be recovered from them by a linear model. Using the vocabulary of all diseases and drugs used in the clinical tasks data sets, we sampled the 2500 most feminine and 2500 most masculine words, based on their relation to the he-she vector, to build a training and test set for the iterative method. We applied the projection process for 35 iterations. Before the process, a linear classifier could determine the gender of the words in the test set with an accuracy of 0.93. The accuracy dropped to 0.37 after the process.

*TeDi-BERT*—the TeDi-BERT model, trained as described in the Implementation Details section.

### Ethical Considerations

All data sets used in this study are previously existing data sets, which are either anonymous or deidentified. The data sets containing clinical trial information (PubMed and ClinicalTrials.gov) are anonymous: they do not contain any single patient data, only aggregated data from all trial participants. The publicly available MIMIC-III data set that we use is deidentified and was approved as part of the original MIMIC-III project [27] by the institutional review boards of Beth Israel Deaconess Medical Center and the Massachusetts Institute of Technology. Therefore, this research did not require additional approval from an ethics committee.

Another ethical consideration is the use of abstracts in the later time range as reference in the optimization function, although they may still contain biases. This may lead to the model having lower performance on diseases where women are still understudied. However, the results described in the next section show improved performance of our method for women, leading us to believe that while this solution is not flawless, it is a step in the right direction toward addressing the effects of bias in clinical word embeddings. More on this in the Limitations section.

## Results

### Hospital LOS Regression

The patient’s diagnoses are given as *ICD-9* (*International Classification of Diseases, Ninth Revision*) codes and mapped into textual descriptions. The sequence of previous diagnoses is embedded using the evaluated embedding model and aggregated using a long short-term memory network (LSTM) layer. The current diagnosis embedding is concatenated to the LSTM output, and demographic features are added. The combined feature vector is fed into a regression model—a 2-layer neural network. The embedding model is frozen, and only the regression model is allowed to train. As the loss function, we use mean square error in the training process and train each model using the Adam optimizer with a learning rate of 1e–3 for 10 epochs (after that, the loss increases).

**Figure 2:**
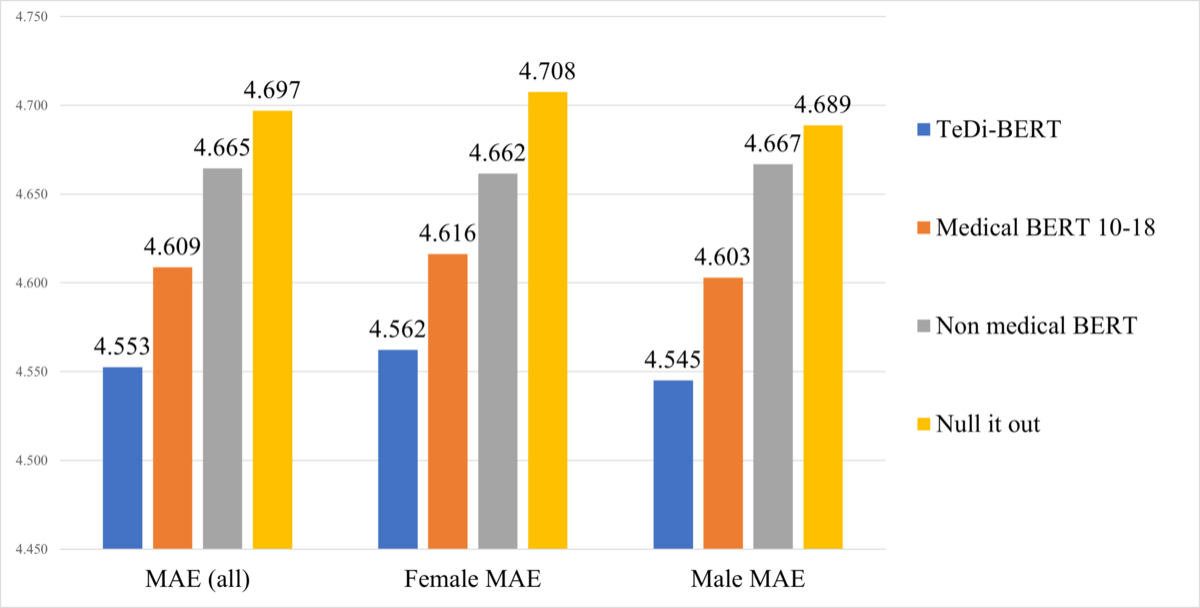
Mean absolute error for LOS regression task using different embeddings. Lower numbers indicate better results. “Null it out” is the work of Ravfogel et al [18]. LOS: length of stay; MAE: mean absolute error.

We report the mean absolute error (MAE) for the compared models, calculated over the entire test set, and aggregated by patient gender (Figure 2 [18]). As expected, the nonmedical BERT does not perform well, as it is not tuned on clinical data. Medical BERT trained in the 2010-2018 range reached better results but applying iterative nullspace projection over medical BERT had lower performance than nonmedical BERT. This can be because the projection alters the embedding space, in the effort to remove gender information; these changes may have harmed the semantic information captured in the embeddings. TeDi-BERT performed best, with a significant improvement in MAE for women and for men (Diebold and Mariano [38] test with mean absolute deviation criterion had *P* value of <.001 for both populations). Further analysis by patient ethnicity (Multimedia Appendix 7) shows that TeDi-BERT performed better than medical BERT over all ethnicity groups but had a specifically large improvement over female patients in minority groups. This suggests that the trends of including underrepresented populations in clinical trials led to the accumulation of a wider knowledge base on these groups. Our model can harness this trend to reach better prediction accuracy on female patients without harming the accuracy on male patients, and even more so in cases of complex bias types, such as gender and race combined. We hypothesize that the performance improvement for men stems from better conduction of clinical trials with relevance to LOS prediction.

### ICU Readmission Prediction

Each element in each of the medications, diagnoses, and previous diagnoses sequences is embedded using the evaluated embedding model. We aggregate the embeddings using an LSTM (with shared weights over the 3 feature sequences). The concatenation of the aggregated embeddings is fed into a classification model (a 2-layer neural network). The models were trained for 4 epochs using the Adam optimizer with a learning rate of 1e–5. The results are measured in area under the receiver operating characteristic curve.

In Lin et al [36], the best model achieved an area under the receiver operating characteristic curve of 0.79, with additional features from the patient medical record events. However, we purposely limited the classifier’s input features to the aforementioned textual fields, since we aim to evaluate the embeddings, and not fully solve the prediction task.

**Figure 3:**
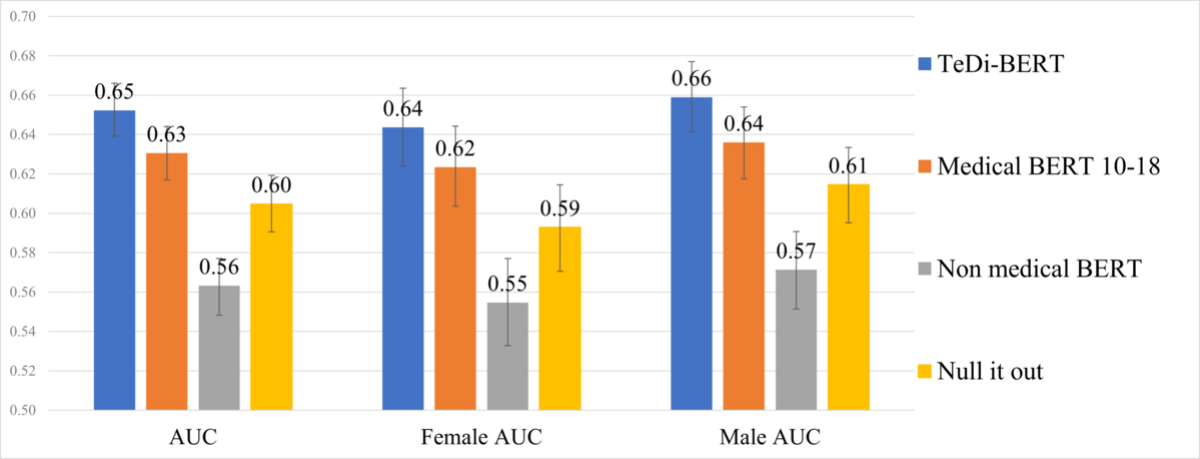
AUC for readmission within 30 days prediction. “Null it out” is the work of Ravfogel et al [18]. AUC: area under the receiver operating characteristic curve.

We analyzed the performance of each model per patient gender (Figure 3 [18]). Further, 95% CIs were calculated using bootstrapping with 2000 resamples over the test set. We further validated the significance of the differences using the DeLong test [39]. All differences for all patient groups were significant with *P*<.001.

As in the previous task, nonmedical BERT results were lower than medical BERT and TeDi-BERT. In this task, applying the debiasing method from Ravfogel et al [18] over medical BERT harmed the performance, but it remained better than nonmedical BERT. TeDi-BERT statistically significantly outperformed all models over female and male patients.

Following the results in the 2 clinical tasks, we conclude that debiasing embeddings through the removal of gender information did not improve the performance on downstream tasks. However, we consistently observe that temporal distribution matching improves performance for female patients.

### Algorithm Analysis

To verify that temporal distribution matching does not harm the semantics learned by the embedding model, we evaluated its quality as a language model. We measured the MLM loss on the validation set of the PubMed corpus (Section S1 in Multimedia Appendix 6). TeDi-BERT’s loss (2.650) was close to that of medical BERT (3.292), indicating that our algorithm maintains the semantic performance of BERT, despite the additional objective of temporal distribution matching. Additionally, we tested the models on named entity recognition tasks (Section S2 in Multimedia Appendix 6) and found that TeDi-BERT did not harm the performance in this task compared to the medical BERT model.

Next, we compared the models on their ability to predict future semantic relatedness of medical concepts, by ranking pairs of medical concepts according to their embedding similarity in each model and comparing the ranking correlation to that of a medical BERT model trained on 2020 abstracts (Section S3 in Multimedia Appendix 6). TeDi-BERT reached the highest-ranking correlation, meaning that TeDi-BERT was able to predict concept similarity from 2020 better than medical BERT, without ever training on texts from 2020. This strengthens our hypothesis that indeed TeDi-BERT can better capture temporal trends in the embeddings, as measured by word similarities, compared to other BERT models.

Additionally, we performed an ablation test, to evaluate the impact of the anchor model in TeDi-BERT (Section S4 in Multimedia Appendix 6). A TeDi-BERT model without an anchor model performed similarly to TeDi-BERT on the MLM task, but its performance on the semantic relatedness task was the lowest of all compared baselines. This shows the necessity of using an anchor model in the training process of distribution matching.

Finally, we used another ablation test to assess the impact of the weight given to old and new abstracts in the training process (Section S5 in Multimedia Appendix 6). We found that a higher weight given to old abstracts caused lower performance in both clinical tasks and the semantic relatedness task. We concluded that indeed matching the older abstracts to the new ones has a positive impact on performance.

### Comparison to Imbalanced Learning Methods

In the MIMIC-III downstream tasks, one could argue that the unbalanced numbers of female (43.97%, 13,553/30,817) and male patients cause a performance gap. We experimented with 3 methods of handling imbalanced data. In all methods, the training set for both tasks was modified to contain 50% women, without modifying the test set.

- Downsampling—downsampling the male patients randomly so that female and male patient numbers are equal (13,553) in the training set.
- Synthetic Minority Over-Sampling Technique (SMOTE) [40]—a classic imbalanced learning method to generate synthetic samples based on neighbors from the same group. We applied SMOTE on the female patients in each downstream task separately and generated 3711 additional samples, so the train set contained 17,264 male patients and 17,264 female patients.
- MedGAN [41]—a widely used synthetic generation method for patient data, that has recently shown promising results in predictive diagnostic tasks. MedGAN combines an autoencoder and a GAN to generate realistic synthetic patient data. For each downstream task, we trained MedGAN on the female patient admissions in the training set and used it to generate additional synthetic admissions, so the train set contained 17,264 male patients and 17,264 female patients.

We trained our prediction models with medical BERT 2010-2018 embeddings on the modified training sets, using the same methods and parameters as in our main results, and compared the results to TeDi-BERT.

**Figure 4:**
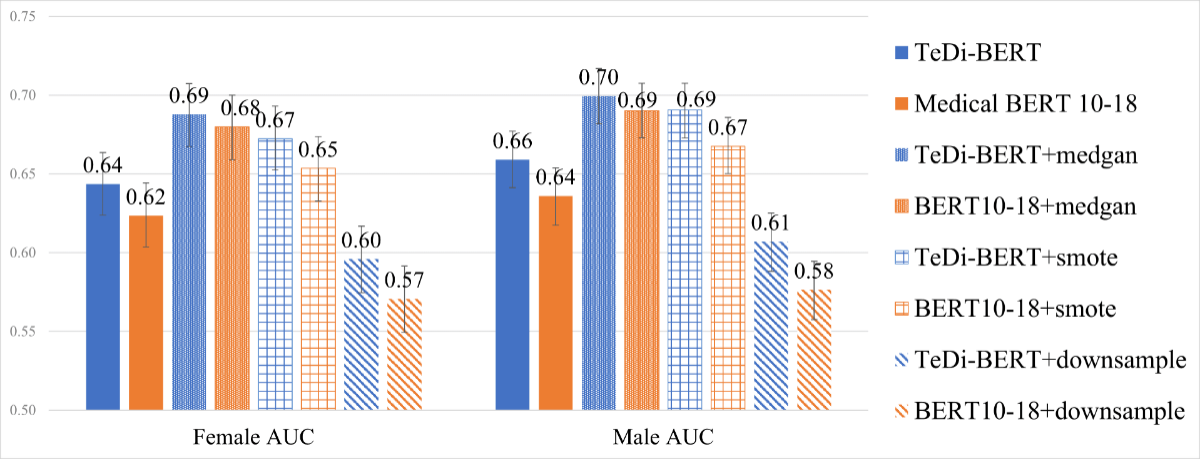
Readmission prediction—comparison of TeDi-BERT versus medical BERT under various methods of handling imbalanced data. The performance is measured in area under the ROC curve, so higher numbers indicate better results. Further, 95% CIs were calculated using bootstrapping with 2000 resamples over the test set. AUC: area under the receiver operating characteristic curve; ROC: receiver operating characteristic curve; TeDi-BERT: temporal distribution matching applied on BERT.

In ICU readmission prediction (Figure 4), downsampling the male patients harmed the performance for both male and female patients and for both models. SMOTE and MedGAN upsampling improved the performance for both populations and both models, but TeDi-BERT still outperformed medical BERT 2010-2018 under MedGAN (*P*=.03 for female patients, *P*=.002 for male patients) and SMOTE (*P*<.001).

**Figure 5:**
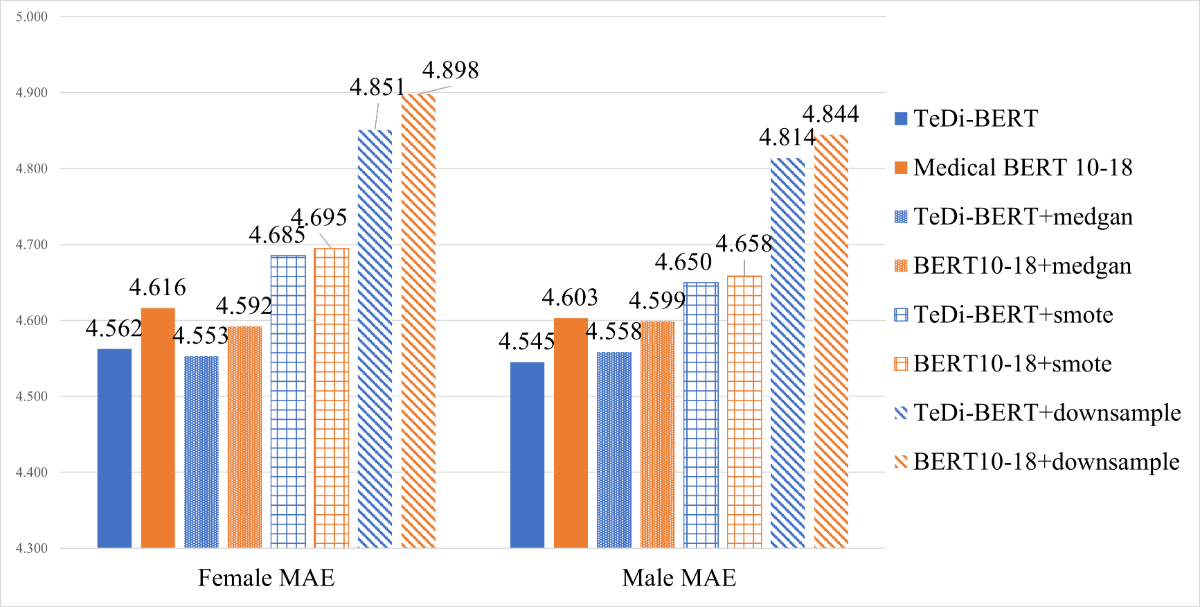
Length of stay prediction—comparison of TeDi-BERT versus medical BERT under various methods of handling imbalanced data. The performance is measured in mean absolute error, so lower numbers indicate better results. MAE: mean absolute error; TeDi-BERT: temporal distribution matching applied on BERT.

In LOS prediction (Figure 5), downsampling and SMOTE upsampling harmed medical BERT’s and TeDi-BERT’s performance, for both patient populations.

MedGAN sampling did not harm the performance, but it did not significantly improve it for either of the models. It is possible that the generated female samples were too noisy to provide added value. Additionally, these methods were designed for much more extreme imbalances than in this setting. This is consistent with several previous works: in multilingual translation [42], upsampling low-resource languages did not robustly improve the loss. In a classification of diseases from textual descriptions of symptoms [43], upsampling rare diseases led to unstable results and in some cases hurt performance.

Over both tested tasks, both populations, and all 3 imbalanced learning methods, TeDi-BERT performed better than medical BERT 2010-2018. We conclude that imbalanced learning techniques may improve performance, but it is not robust to all tasks and models. As with many other possible techniques to improve performance (data cleaning, feature engineering, etc), imbalanced learning techniques may be applied independently from the choice of embedding model.

## Discussion

### Principal Results

In both clinical tasks, TeDi-BERT’s performance for female patients was significantly improved compared to medical BERT 2010-2018, while improving performance on male patients as well. This is even though both models were trained on the same data set of clinical trial abstracts. The advantages of the TeDi-BERT method were especially large for population groups subject to intersectional biases (Multimedia Appendix 7), which suggests that other than gender inclusion, additional improvement trends in clinical trials were captured by the TeDi-BERT model. When analyzing the contribution of our method for different feature types in the LOS task (Multimedia Appendix 8), we found that for both models, the primary diagnosis was more predictive of the LOS than the previous diagnoses, but TeDi-BERT was able to use the information in previous diagnoses to reduce the MAE more than medical BERT 2010-2018.

A baseline debiasing method based on the removal of gender information from word embeddings [18] did not perform well in the clinical prediction tasks, achieving worse results than medical BERT 2010-2018. This validates our hypothesis that the removal of information about a sensitive attribute from the embeddings is not a suitable strategy for debiasing medical embeddings since that sensitive attribute contains valuable clinical information.

In the semantic task of MLM (Section S1 in Multimedia Appendix 6), TeDi-BERT’s performance surpassed that of medical BERT 2010-2018, despite the competing objective functions of the generator and the discriminator. In another semantic task based on temporal trends (Section S3 in Multimedia Appendix 6), while both models were trained on the same data set, TeDi-BERT’s output was more similar to that of a model trained only on clinical trials from 2020. This validates our hypothesis that TeDi-BERT is better at capturing the temporal trends in the data than medical BERT 2010-2018.

When comparing TeDi-BERT to various imbalanced learning methods, we found that temporal distribution matching had a consistent contribution to performance, while imbalanced learning methods harmed performance in some cases.

When comparing TeDi-BERT to gender-sensitive weighting of the corpus (Multimedia Appendix 2), we found that gender-sensitive weighting was not a good fit for debiasing BERT embeddings for health care, despite its success for Word2vec embeddings. We hypothesize that this is due to the complexity of the BERT embedding model versus Word2vec and that a finer method is required for debiasing BERT embeddings.

The empirical results show the merit of debiasing embeddings for improving the performance of clinical tasks. Despite the remaining biases in the newer clinical trials, leveraging the temporal trends of bias reduction was successful for the reduction of biases in the embeddings.

Although many works show the trade-off between fairness and accuracy [44-46], our results show that accuracy for one gender does not need to be exchanged for bias reduction, but rather that good science improves clinical results for all.

### Limitations

Our work has several limitations. In our TeDi-BERT implementation, we divided clinical trials into 2 time ranges (old and new). This approach is inspired by related work in adversarial domain adaptation [26], where there is a source and target domain. For future work, we wish to expand the approach to a continuous prediction. Additionally, the temporal distribution matching might obfuscate temporal markers such as new diseases or treatments; this can be mitigated by the development of techniques to handle out-of-vocabulary words. Finally, another limitation is the remaining biases in recent clinical trials and the continued underrepresentation of women in them. The use of a still-biased data distribution as the optimization target may cause difficulties in the categorization of diseases where women are still not studied enough, because the knowledge captured in the word embeddings about these conditions may still be partial. However, in many diseases (eg, cardiovascular diseases, anemia, osteoporosis, and more) the situation has greatly improved in recent years. As a result, TeDi-BERT achieved higher performance and lower gender performance gaps in the tested clinical tasks. While it is not a perfect solution, the experimental results show that it is in the correct direction toward fixing the problem. We believe that temporal distribution matching is a good proxy for bias mitigation, but more direct approaches should also be tested.

### Conclusions

The use of clinical trials as a training corpus for embedding models should be conducted with care while taking precautions against the long-existing biases in clinical trials. We presented TeDi-BERT, a method for training word embeddings while harnessing a temporal trend in the corpus. The method includes a novel use of the GAN framework to regularize for temporal distribution matching on embedded samples. We implemented our method on BERT, a contextual embedding model that achieved state-of-the-art results in many NLP tasks, and trained it on clinical trial abstracts, where biases, and especially enrollment gender bias, are reduced over time for a significant portion of researched concepts. In our experimental evaluation, we demonstrated performance improvement over BERT in clinical prediction tasks. We found that the performance particularly improved for female patients for all tasks, and for male patients either improved or did not harm performance. This suggests that adjusting for bias in research can benefit clinical results for all patients.

## Appendix

Multimedia Appendix 1 Analysis of chosen time periods.

Multimedia Appendix 2 Comparison to gender-sensitive debiasing.

Multimedia Appendix 3 Qualitative analysis of trends in clinical trials.

Multimedia Appendix 4 Comparison to larger BERT models.

Multimedia Appendix 5 TeDi-BERT technical details. TeDi-BERT: temporal distribution matching applied on BERT.

Multimedia Appendix 6 Algorithm analysis.

Multimedia Appendix 7 Analysis of length of stay performance by gender and ethnicity.

Multimedia Appendix 8 Ablation—feature set contribution.

## Abbreviations

GAN: generative adversarial network
ICD-9: International Classification of Diseases, Ninth Revision
ICU: intensive care unit
LOS: length of stay
LSTM: long short-term memory network
MAE: mean absolute error
MLM: masked language modeling
NLP: natural language processing
SMOTE: Synthetic Minority Over-Sampling Technique
TeDi-BERT: temporal distribution matching applied on BERT

## Data Availability

The PubMed data is publicly available [34]. The clinical trials metadata is publicly available [35]. MIMIC-III is publicly available [47] pending the completion of a training course.
