# Supplementary material for: Leveraging Temporal Trends for Training Contextual Word Embeddings to Address Bias in Biomedical Applications: Development Study
Source: JMIR AI. 2024 Oct 2;3:e49546. doi: 10.2196/49546 (PMC11483253; doi:10.2196/49546)
Supplement: Multimedia Appendix 1 [file ai_v3i1e49546_app1.docx]

To validate our choice of old and new abstracts, we compared the percentage of female participants in clinical trials in the two time periods. We restricted the analysis to clinical trials that were associated with common disease MeSH terms. To choose the terms, we traversed the MeSH taxonomy tree and selected the top-level terms under “Diseases”. We filtered out terms that were mentioned in less than 100 clinical trials in each of the chosen time ranges. We used the remaining terms to filter the clinical trials according to their PubMed abstracts.

When summing all the female and male participants in the clinical trials in those years, we found that the total percent of female participants was 0.48 in 2010-13 versus 0.51 in 2016-18, which a statistically significant difference, verified through a two-proportion two-tailed Z-test (Z=-244.5, p-value=0). When averaging the female percentages of clinical trials, the mean was also increased (0.48 in 2010-13 versus 0.49 in 2016-18), which is also statistically significant (2 tailed T-test statistic=-2.01, p-value=0.04).

The improvement in women's representation between the two time periods can lead to discoveries that are less biased towards women. This can be seen through changes in relations between concept embeddings over time. We compared BERT embeddings based on 2010-13 clinical trials versus 2016-18. We found that cosine similarity was increased between ``Chronic Obstructive Pulmonary Disease'' and ``Dyspnea'', a comorbidity of COPD more prevalent in women [1]. Cosine similarity was decreased between the term ``women'' and the terms ``Anorexia'' and ``Mutism'', which are mental health concepts that were found to be biasedly associated with women [2].

## References

1. Perez TA, Castillo EG, Ancochea J, et al. Sex differences between women and men with COPD: A new analysis of the 3CIA study. Respiratory medicine 2020;171:106105. PMID:32858497
2. Rios A, Joshi R, and Shin H. Quantifying 60 Years of Gender Bias in Biomedical Research with Word Embeddings. Proceedings of the 19th SIGBioMed Workshop on Biomedical Language Processing, 2020 July; Online. Association for Computational Linguistics; 2020. doi:10.18653/v1/2020.bionlp-1.1
