# Supplementary material for: Leveraging Temporal Trends for Training Contextual Word Embeddings to Address Bias in Biomedical Applications: Development Study
Source: JMIR AI. 2024 Oct 2;3:e49546. doi: 10.2196/49546 (PMC11483253; doi:10.2196/49546)
Supplement: Multimedia Appendix 2 [file ai_v3i1e49546_app2.docx]

A previously proposed method for debiasing of word embeddings in healthcare [1] used data augmentation to mitigate the effects of the underrepresentation of women. Each clinical trial abstract was weighted according to the number of female participants in the trial, and the word embedding model was trained on the modified corpus. The method was shown to be effective for Word2Vec [2] word embeddings, increasing the performance over several clinical tasks.

We compared the performance of our method to gender-sensitive weighting of the corpus. Each abstract was given an importance score as described in [1] and repeated several times according to that score. We then trained BERT-Tiny [3] on the modified corpus for 3 epochs and tested its performance on the various tasks. Note that the repetitions of the abstracts increased the corpus by approximately 14 times; therefore, a smaller number of epochs was required to reach a saturation in the MLM loss.

The masked language modelling loss was 2.305, lower than the MLM loss for the neutral (non-gender sensitive) Medical BERT 2010-18 and for TeDi-BERT. This suggests that 3 epochs were enough to train the model. In the semantic relatedness task (Section S3 in Multimedia Appendix 6), gender-sensitive BERT achieved a correlation of 0.73 with Medical BERT 2020; higher than non-medical BERT (0.45), similar to Medical BERT 2010-18 (0.74) and lower than TeDi-BERT (0.78). In the clinical task of readmission prediction, gender-sensitive BERT achieved an AUC of 0.632 for female patients and 0.653 for male patients, which is better than Medical BERT but not as good as TeDi-BERT (*P*<0.001 for women, *P*=0.014 for men). Finally, in the length of stay prediction task, gender-sensitive BERT’s MAE was 4.743 for women and 4.762 for men, which is worse than Medical BERT and TeDi-BERT.

We conclude that gender-sensitive weighting was not a good fit for debiasing BERT embeddings for healthcare, despite its success for Word2Vec embeddings. The BERT embedding model is more complex than Word2Vec; it considers the context of each word (the other words in the sentence) when transforming a word into its representation. We hypothesize that simple reweighting of the corpus had an effect which was too strong on the contextualized BERT model, and a finer method is required for debiasing BERT.

## References

1. Agmon S, Gillis P, Horvitz E, et al. Gender-sensitive word embeddings for healthcare. Journal of the American Medical Informatics Association, 2022;29(3):415-423. doi:10.1093/jamia/ocab279
2. Mikolov T, Chen K, Corrado G et al. Efficient estimation of word representations in vector space. arXiv preprint arXiv:1301.3781, 2013. doi:10.48550/arXiv.1301.3781
3. "Google BERT uncased L2 H128 A2," 2019. [Online]. Available: https://huggingface.co/google/bert_uncased_L-2_H-128_A-2. [Accessed 14 February 2023].
