# Supplementary material for: Leveraging Temporal Trends for Training Contextual Word Embeddings to Address Bias in Biomedical Applications: Development Study
Source: JMIR AI. 2024 Oct 2;3:e49546. doi: 10.2196/49546 (PMC11483253; doi:10.2196/49546)
Supplement: Multimedia Appendix 3 [file ai_v3i1e49546_app3.docx]

To analyse the temporal trends in clinical trials, we employed topic modeling, using BERTopic [1]. We first filtered the clinical trial abstracts according to their MeSH terms, to find abstracts about a specific disease. Then we applied BERTopic on the filtered abstracts, to extract the most prominent subtopics.

BERTopic is a deep learning method to perform topic modeling of a set of documents. It maps each document to an embedding. It then performs dimensionality reduction and groups the vector representations into clusters. Each cluster corresponds to a topic found in the text. Finally, each cluster is characterized by the distribution of words in the documents that belong to the cluster, and the most typical words for each cluster are chosen to represent the topic. The trained topic model can then be used to map a given document to the distribution of topics covered in it. We used KeyBERT [2]-inspired representation and minimal cluster size of 10 and left the other parameters at their default setting.

After training and applying BERTopic on the abstracts matching the MeSH term, we inspected the frequency of each subtopic over time and matched a linear regression line to it. Finally, we analysed the subtopics with the largest change in frequency over time, which means the largest absolute value of regression coefficient.

The first disease we analyzed is Type 2 Diabetes Mellitus – with a total of 3199 clinical trial abstracts until 2020. The changes over time for 3 subtopics are shown in figure S1.


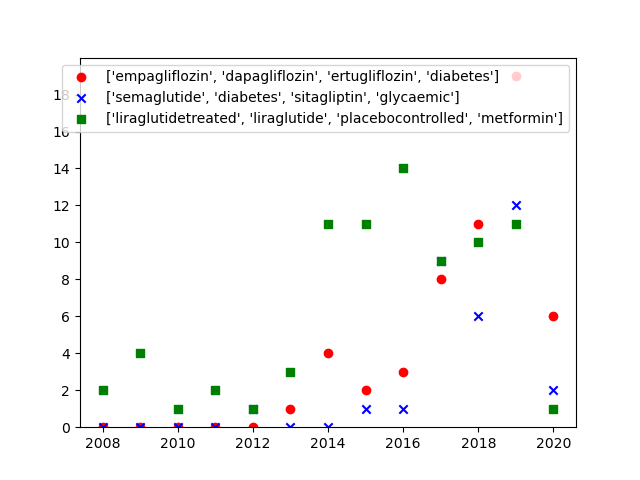


Figure S1: Trends in Type 2 Diabetes Mellitus subtopics presented as abstract frequency over time.

The first topic (red circles) contains several drug names, which are all

Sodium-glucose Cotransporter-2 inhibitors. The sudden rise in abstract frequency around 2014 can be explained by the FDA approval of Empaglifozin [3] and Dapaglifozin [4] which were both in that year.

The second topic (blue Xs) contains the names of two drugs which increase insulin levels by targeting the GLP-1 hormone: Semaglutide mimics its action [5], while Sitagliptin slows down the breakdown of GLP-1 [6]. The rise in abstract frequency around 2018 is perhaps due to FDA approval of Semaglutide in late 2017 [5].

The third topic (green squares) is a drug called Liraglutide, whose FDA approval for adults with BMI>27 in 2014 [7] is likely the cause of the increase in its popularity in clinical trials in that year.

The second disease we analyzed is Myocardial Infarction, with a total of 1833 clinical trial abstracts until 2020. The changes in 3 of its subtopics are presented in Figure S2.


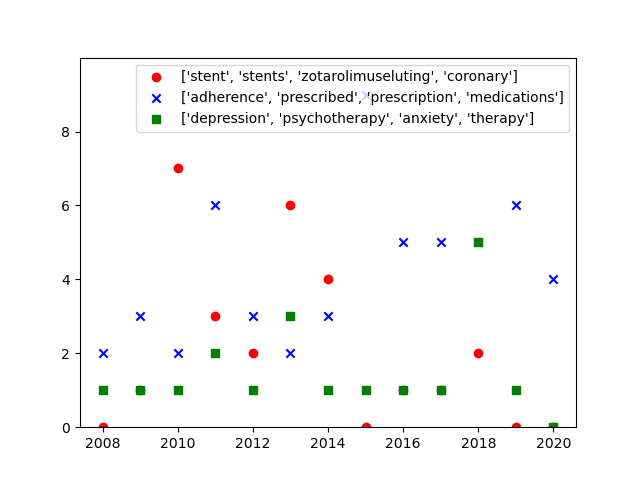


Figure S2: Trends in Myocardial Infarction subtopics presented as abstract frequency over time.

The first topic (red circles) keywords describe stents, a process to hold open blocked arteries [8]. There is a decline in the frequency of abstracts on this topic, which is consistent with recent clinical trials (ORBITA [9], ISCHEMIA [10], REVIVE [11]) that found no difference in outcomes for stents versus drug therapy.

In parallel to the decrease in stent popularity in clinical trials, we see an increase in clinical trials about adherence to prescribed medications (blue Xs), which are an alternative treatment to stents. Finally, there is a slight increase in the frequency of clinical trials about psychological aspects of patients with MI (green squares).

This qualitative analysis shows that real world events have a direct and immediate impact on clinical trial abstracts, and temporal trends are present and detectable in the data.

## References

1. Grootendorst M. BERTopic: Neural topic modeling with a class-based TF-IDF procedure. arXiv preprint arXiv, vol. 2203.05794, 2022. doi: 10.48550/arXiv.2203.05794
2. Grootendorst M. KeyBERT: Minimal keyword extraction with BERT, 2020. doi:10.5281/zenodo.4461265
3. Fala L. Jardiance (Empagliflozin), an SGLT2 Inhibitor, Receives FDA Approval for the Treatment of Patients with Type 2 Diabetes. Am Health Drug Benefits 2015;8:92-95. PMID: [26629271](https://pubmed.ncbi.nlm.nih.gov/26629271)
4. Golderberg MM. Pharmaceutical Approval Update. Pharmacy and Therapeutics 2014;39:3. PMID:24790394
5. Development Status and FDA Approval Process for semaglutide. Drugs.com, [Online]. Available: https://www.drugs.com/history/ozempic.html. [Accessed October 2023].
6. "Januvia EPAR," European Medicines Agency, 2018. [Online]. Available: https://www.ema.europa.eu/en/medicines/human/EPAR/januvia. [Accessed October 2023].
7. "Drug Approval Package: Saxenda Injection (Liraglutide [rDNA origin])," U.S. Food and Drug Administration (FDA), [Online]. Available: https://www.accessdata.fda.gov/drugsatfda_docs/nda/2014/206321Orig1s000TOC.cfm. [Accessed October 2023].
8. "Stents - What Are Stents?," NHLBI, NIH, 2022. [Online]. Available: https://www.nhlbi.nih.gov/health/stents. [Accessed October 2023].
9. Al-Lamee R, Thompson D, Hakim-Moulay D et al. Percutaneous coronary intervention in stable angina (ORBITA): a double-blind, randomised controlled trial. Lancet 218;391(10115):31-40. PMID:29103656
10. P. Wendling, "ISCHEMIA: PCI, Surgery Strike Out vs Meds," Medscape, 16 November 2019. [Online]. Available: https://www.medscape.com/viewarticle/921463. [Accessed October 2023].
11. Perera D, Clayton T, O'kane P, et al. Percutaneous Revascularization for Ischemic Left Ventricular Dysfunction. N Engl J Med 2022;387(15):1351-1360.
