# Supplementary material for: Leveraging Temporal Trends for Training Contextual Word Embeddings to Address Bias in Biomedical Applications: Development Study
Source: JMIR AI. 2024 Oct 2;3:e49546. doi: 10.2196/49546 (PMC11483253; doi:10.2196/49546)
Supplement: Multimedia Appendix 4 [file ai_v3i1e49546_app4.docx]

We trained BERT-base-uncased [1] on the same data as Medical BERT 2010-2018 for 40 epochs.

We compared the performance of this model versus the tiny BERT model trained on the same data, in the hospital length of stay prediction task. The RMSE of Medical BERT (base) was the same as for Medical BERT (tiny): 6.04. Therefore, we concluded that there is no advantage to using the larger BERT models in this case.

One might think that the improvement seen in the clinical tasks (Hospital Length of Stay Regression and ICU Readmission Prediction sections) for TeDi-BERT over Medical BERT could be due to a larger number of parameters in TeDi-BERT. However, the result above shows that it is unlikely: simply increasing the model size does not always improve the results. Furthermore, the additional parameters in TeDi-BERT are frozen, and the representation size is the same in both models.

## References

1. "BERT-base-uncased - Hugging Face," [Online]. Available: https://huggingface.co/bert-base-uncased. [Accessed December 2023].
