# Supplementary material for: Leveraging Temporal Trends for Training Contextual Word Embeddings to Address Bias in Biomedical Applications: Development Study
Source: JMIR AI. 2024 Oct 2;3:e49546. doi: 10.2196/49546 (PMC11483253; doi:10.2196/49546)
Supplement: Multimedia Appendix 5 [file ai_v3i1e49546_app5.docx]

In our experiments (Results section), each BERT model contains 4.4M parameters, and TeDi-BERT contains 4.4M trainable parameters and 4.4M frozen parameters of the anchor model. The resulting embedding size is 128 in all compared models, including TeDi-BERT. Each clinical prediction model described in that section contains 4.6M parameters. The models required 11 GPU training hours in total. All models were trained on a single NVIDIA GeForce GTX 1080 GPU.
