# Supplementary material for: Leveraging Temporal Trends for Training Contextual Word Embeddings to Address Bias in Biomedical Applications: Development Study
Source: JMIR AI. 2024 Oct 2;3:e49546. doi: 10.2196/49546 (PMC11483253; doi:10.2196/49546)
Supplement: Multimedia Appendix 6 [file ai_v3i1e49546_app6.docx]

### Section S1 **Embedding Quality as a Language Model**

To verify that temporal distribution matching does not harm the semantics learnt by the embedding model, we evaluate its quality as a language model. We measure the masked language modeling (MLM) loss on the validation set of the PubMed corpus.

The non-medical BERT had the highest loss (5.389); this is expected, because it was not trained on medical texts. The medical BERT loss was lower (3.292), and TeDi-BERT’s loss was lower still (2.650), indicating that TeDi-BERT is doing well in the MLM task on the corpus. We can conclude that our algorithm maintains the semantic performance of BERT, despite the additional objective of temporal distribution matching.

### Section S2 **Embedding Quality in Named Entity Recognition**

To verify that the TeDi-BERT method does not harm the usefulness of the embedding, we tested the performance over the common NLP task of named entity recognition (NER). We used two benchmark datasets: NCBI-disease [1] (6,881 annotations) and BCD5CDR-disease [2] (12,694 annotations). On each of them, we compared the performance of three models: non-medical BERT, medical BERT 2010-2018 and TeDi-BERT. For this task we initialized the models from bert-base-uncased. The medical BERT model was trained for 40 epochs over the clinical trial corpus. For the TeDi-BERT model we used the medical BERT model as anchor and trained it for 20 epochs with learning-rate 2e-5 with $\lambda_{A}=0.3, \lambda_{adv}=0.3$. On the downstream task, the models were fine-tuned with learning-rate 1e-4 for 4 and 5 epochs on the BCD5CDR and NCBI tasks, respectively. The results are shown in Table S1.

Unlike the clinical tasks shown in the Results section of the paper, the NER tasks do not contain a protected attribute (e.g., gender or race) or an aspect of bias towards a protected group. Therefore, we did not expect the TeDi-BERT model to have an advantage over the Medical BERT model. Instead, the goal of this experiment is to verify that temporal distribution matching does not harm the usability of the model in common NLP tasks. Indeed, the differences among the models are very small, and there is no clear advantage to one model over the others. The results are comparable to those achieved by BioBERT [3] which was trained longer and on more PubMed abstracts. This suggests that the TeDi-BERT method does not harm the usability of the embeddings on standard NLP tasks.

|  | Metric | NCBI | BCD5CDR |
| --- | --- | --- | --- |
| Non-medical BERT | P | 0.8611 | 0.8113 |
|  | R | 0.8912 | 0.8668 |
|  | F | 0.8759 | 0.8382 |
| Medical BERT 2010-2018 | P | 0.8632 | 0.8473 |
|  | R | 0.8836 | 0.8603 |
|  | F | 0.8733 | 0.8538 |
| TeDi-BERT | P | 0.8624 | 0.8406 |
|  | R | 0.8836 | 0.8600 |
|  | F | 0.8729 | 0.8502 |

Table S1: Evaluation results of the embeddings over NER tasks.

### **Section S3 Predicting Future Semantic Relatedness of Medical Concepts**

Semantic relatedness is a common method of evaluating word embeddings [4-6] (and many others), where the similarity between word embedding pairs is compared to a gold standard created by manual annotations. Previous works [7-8] researched the changes in text corpora over time through temporal semantic relatedness: the similarities between concepts in language models trained over different time periods. We draw from this idea, to evaluate the ability of the compared models to capture temporal trends. We create a golden ranking of concept pairs, according to their similarity in Medical BERT 2020. If a language model is able to capture temporal trends well, we expect it to be able to predict future similarities; i.e., we expect that the ranking of the concept pairs according to their similarity in this model to be similar to the golden (2020) ranking.

Therefore, we ranked the concept pairs by their embedding similarity in each evaluated model, and computed the Spearman correlation with the golden ranking. We evaluated this metric for Medical BERT 2010-2018 and TeDi-BERT and added Medical BERT 2018 and Non-medical BERT for comparison. The results are presented in Table S2. All correlations are statistically significant.

Unsurprisingly, Non-medical BERT had the lowest ranking correlation with BERT-2020 (0.45), due to the difference in domains. Medical BERT, trained on clinical trials from 2010-2018 without modification, produced a considerably higher correlation (0.74). The results are higher than Medical BERT 2018 (0.55), indicating that temporal trends over a longer period of time are important to capture temporal semantics. TeDi-BERT reached the highest correlation of 0.78, meaning that TeDi-BERT was able to predict concept similarity from 2020 better than Medical BERT, without ever training on texts from 2020. This strengthens our hypothesis that indeed TeDi-BERT is able to better capture temporal trends in the embeddings, as measured by word similarities, compared to other BERT models.

| **Model** | **Ranking Correlation with 2020** |
| --- | --- |
| Non-Medical BERT | 0.45 |
| Medical BERT 2018 | 0.55 |
| Medical BERT 2010-2018 | 0.74 |
| TeDi-BERT | 0.78 |

Table S2: Comparison of the models to BERT trained only on clinical trials from 2020. We compare the Spearman correlation of ranking concept pairs by their similarity.

### Section S4 The Impact of an Anchor Model

We used an ablation test to evaluate the impact of the comparison to a frozen anchor model. We trained a model of the same architecture as TeDi-BERT but set $\lambda_{A}=0$, so that the distance from the anchor model will not affect the loss function. We set $\lambda_{D}=0.5$, to give an equal weight to the MLM loss and the negated discriminator loss. The model was trained for 20 epochs, like the TeDi-BERT model we used in previous evaluations. We denote this model non-anchored TeDi-BERT.

In the MLM task, non-anchored TeDi-BERT’s loss (2.91) was slightly higher than TeDi-BERT but lower than medical BERT. This is expected, as both non-anchored TeDi-BERT and TeDi-BERT have the MLM loss component in their loss function and were trained specifically on the PubMed corpus.

However, when testing the ranking correlation with Medical BERT 2020 (Appendix 6.3), non-anchored TeDi-BERT performed worse than all other models (correlation=0.39), including non-medical BERT. This shows the necessity of using an anchor model in the training process of distribution matching.

### Section S5 Ablation: Impact of Old and New Abstracts on Performance

To assess the impact of old and new clinical trials, we added a weight parameter $\alpha$ to the binary cross entropy formula, which is the adversarial loss function:

$$\min_{\theta_{M_{e}}} \max_{\theta_{M_{d}}} L_{\mathrm{adv}} ={\left( 1-\alpha\right)\mathbb{\cdot E}}_{A_{i}\in A_{\mathrm{new}}}[log(M_{d}(M_{e}(A_{i})))]+{\alpha\cdot\mathbb{E}}_{A_{i}\in A_{\mathrm{old}}}[1-log(M_{d}(M_{e}(A_{i})))]$$

If $\alpha=0$, the discriminator causes the newer sample distribution to drift towards the old sample distribution. The TeDi-BERT model used in our experiments is equivalent to setting $\alpha=0.5$, and we trained an additional model with $\alpha=0.2$. We compared the performance of the three models on the tasks of length of stay and readmission prediction for female patients, and semantic relatedness to the abstracts of 2020 (Figure S3).


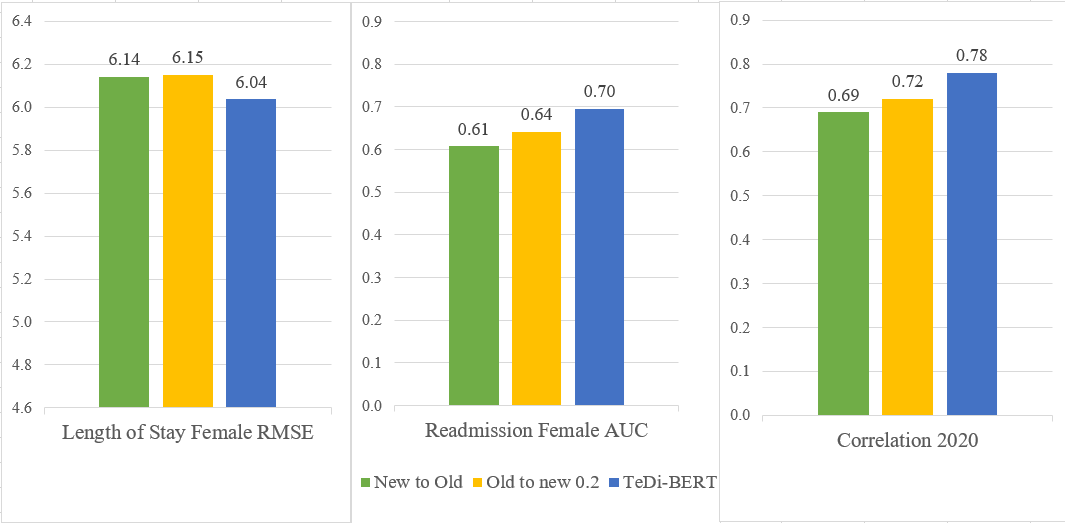


Figure S3: Comparison of varying weightings of old and new samples in length of stay, readmission prediction, and ranking correlation with 2020.

In LOS, the two models with $\alpha<0.5$ reached a higher RMSE loss than TeDi-BERT. This was also the case for readmission prediction for female patients: the AUC was highest for TeDi-BERT (0.7). This pattern also repeated in the semantic relatedness task (the highest Pearson correlation with BERT 2020 was 0.78 for $\alpha=0.5$, and lower for $\alpha<0.5$). We conclude that indeed matching the older abstracts to the new has a positive impact on performance.

## References

1. Doğan RI, Leaman R and Lu Z. NCBI disease corpus: a resource for disease name recognition and concept normalization. Journal of biomedical informatics 2014;47:1–10. PMID:24393765
2. Li J, Sun Y, Johnson RJ, et al. BioCreative V CDR task corpus: a resource for chemical disease relation extraction. Database 2016;2016. PMID:27161011
3. Lee J, Yoon W, Kim S, et al. BioBERT: a pre-trained biomedical language representation model for biomedical text mining. Bioinformatics 2020;36:1234–1240. doi:10.1093/bioinformatics/btz682
4. Finkelstein L, Gabrilovich E, Matias Y, et al. Placing search in context: The concept revisited. Proceedings of the 10th international conference on World Wide Web; 2001 May 1-5; Hong Kong, China.
5. Liu Y, McInnes BT, Pedersen T, et al. Semantic relatedness study using second order co-occurrence vectors computed from biomedical corpora, UMLS and WordNet. Proceedings of the 2nd ACM SIGHIT International Health Informatics Symposium; 2012 January 28-30; New York, NY, United States. doi:10.1145/2110363.2110405
6. Luong MT, Socher R and Manning CD. Better word representations with recursive neural networks for morphology. Proceedings of the seventeenth conference on computational natural language learning, 2013 August; Sofia, Bulgaria. Association for Computational Linguistics.
7. Kim Y, Chiu YI, Hanaki K, et al. Temporal Analysis of Language through Neural Language Models. Proceedings of the ACL 2014 Workshop on Language Technologies and Computational Social Science, 2014 June; Baltimore, MD, United Stated. Association for Computational Linguistics. doi:10.3115/v1/W14-2517
8. Rosin GD, Adar E and Radinsky K. Learning Word Relatedness over Time. Proceedings of the 2017 Conference on Empirical Methods in Natural Language Processing, 2017 September 7-11; Copenhagen, Denmark. Association for Computational Linguistics. doi:10.18653/v1/D17-1121
