# Supplementary material for: Leveraging Temporal Trends for Training Contextual Word Embeddings to Address Bias in Biomedical Applications: Development Study
Source: JMIR AI. 2024 Oct 2;3:e49546. doi: 10.2196/49546 (PMC11483253; doi:10.2196/49546)
Supplement: Multimedia Appendix 7 [file ai_v3i1e49546_app7.docx]

To further analyze the results for length of stay prediction, we divided the patients in the test set by ethnicity and gender. The ethnicity field in the MIMIC-III dataset contains many different values, and some are very rare, so we grouped them according to the main ethnicity (for example, "ASIAN - VIETNAMESE" and "ASIAN - FILIPINO" were both grouped under "ASIAN"). We included only grouped ethnicity values with at least 30 patients and removed the "OTHER" and "UNKNOWN" categories. For each remaining ethnicity, we calculated the MAE for two models: TeDi-BERT and the medical BERT 2010-2018 baseline. The results are shown in Figure S4.


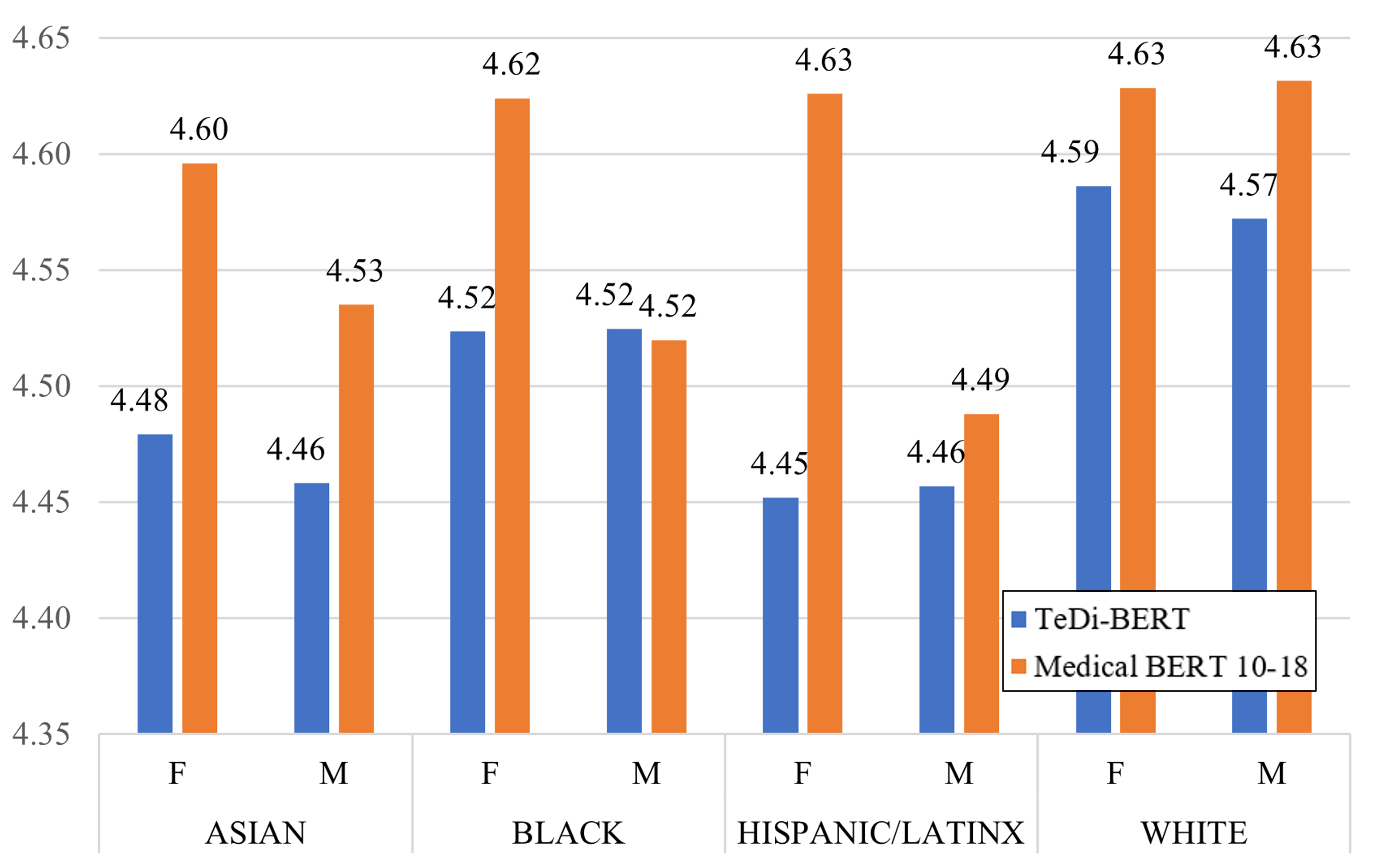


Figure S4 Comparison of Mean Absolute Error for LOS regression task with TeDi-BERT versus Medical BERT, analyzed by gender and ethnicity.

We observed that for all races and genders, the TeDi-BERT model performed similarly to or better than Medical BERT. However, for female patients in the minority groups (Asian – 2.3%, Black – 9.1% and Latinx – 3.6%, vs. 71.9% White for comparison), there was a specifically large advantage to the TeDi-BERT model.

This suggests that the TeDi-BERT model was able to harness the trend of including underrepresented populations in clinical trials and use it to alleviate not only gender bias, but also for other types of bias, including intersectional biases. However, a more detailed analysis of the types of bias is left for future work.
