# Supplementary material for: Leveraging Temporal Trends for Training Contextual Word Embeddings to Address Bias in Biomedical Applications: Development Study
Source: JMIR AI. 2024 Oct 2;3:e49546. doi: 10.2196/49546 (PMC11483253; doi:10.2196/49546)
Supplement: Multimedia Appendix 8 [file ai_v3i1e49546_app8.docx]

To evaluate the contribution of the TeDi-BERT methodology on each feature set, we performed an ablation study on the Length of Stay prediction task, where the clinical prediction model is only given a subset of the features. The textual features are embedded using the trained TeDi-BERT or the Medical BERT model.

Table S3 contains the mean absolute error for female and male patients. Three models are shown: the original model – trained on all features, a model trained only on previous diagnoses, and a model trained only on the primary diagnosis. All models were also given the same demographic and summary features as described for the main results. The ablation models were trained for 10 epochs, like the original model.

| **Features** | **Female patients MAE** | **Male patients MAE** |
| --- | --- | --- |
| (1) All (original), TeDi-BERT | 4.562 | 4.545 |
| (2) All (original), Medical BERT 10-18 | 4.616 | 4.603 |
| (3) Previous diagnoses, TeDi-BERT | 4.748 | 4.758 |
| (4) Previous diagnoses, Medical BERT 10-18 | 4.748 | 4.767 |
| (5) Primary diagnosis, TeDi-BERT | 4.610 | 4.578 |
| (6) Primary diagnosis, Medical BERT 10-18 | 4.588 | 4.565 |

Table S3 Mean absolute error in length of stay prediction with ablation of feature sets, analyzed by patient gender.

For the TeDi-BERT model, both ablations harmed the performance, but removing the primary diagnosis (row 3) was worse than removing the previous diagnoses (row 5) from the input to the regression model. Removing the primary diagnoses harmed performance for Medical BERT 10-18 as well (row 4). This suggests that the primary diagnosis that a patient is given on admittance to the hospital is more predictive of the patient’s length of stay than their medical history.

The TeDi-BERT based model was able to use the information in previous diagnoses to reduce the MAE (row 1) compared to only using the primary diagnoses (row 5), but the Medical BERT 10-18 model did not benefit from the addition of previous diagnoses (rows 2 and 6). Overall, the best performing model was TeDi-BERT with all feature sets included (row 1).
